# Retrospective Case Analysis of Transnasal Endoscopic Resection of Benign Orbital Apex Tumors: Some Thoughts on Transnasal Endoscopic Surgery

**DOI:** 10.1155/2021/6691203

**Published:** 2021-02-13

**Authors:** Cheng Li, Yang Gao, Rongxin Chen, Chao Cheng, Pan Yin, Zhihui Zhang, Yinghao Wang, Yuekun Bao, Huan Ma, Jianbo Shi, Rong Lu

**Affiliations:** ^1^Department of Orbital Diseases and Ocular Oncology, State Key Laboratory of Ophthalmology, Zhongshan Ophthalmic Center, Sun Yat-Sen University, Guangzhou 510060, China; ^2^Department of Otolaryngology, The First Affiliated Hospital, Sun Yat-Sen University, Guangzhou 510080, China

## Abstract

**Purpose:**

To deeply discuss the patient selection, surgical planning, surgical techniques, and the therapeutic challenge for endoscopic transnasal resection of benign orbital apex tumors (OATs).

**Methods:**

We retrospectively analyzed the cases of 18 patients (18 eyes) with orbital apex cavernous hemangioma (OACH) who underwent endoscopic transnasal approach for resection of the tumor in Zhongshan Ophthalmic Center from March 2016 to May 2020. At each follow-up visit, the patients underwent measurement of their best-corrected visual acuity (BCVA), slit-lamp examination, indirect ophthalmoscopy, and visual field testing.

**Results:**

There were 18 patients, 7 males and 11 females, with a mean age of 49.9 ± 12.6 years (range: 26 to 70 years). All 18 patients had unilateral tumors. Among the 18 cases, 13 were located in the right orbit and 5 were located in the left orbit. Sixteen patients underwent purely endoscopic transnasal surgery, and the other 2 patients underwent an endoscopic transnasal approach combined with a transcutaneous or transconjunctival surgical approach. Fourteen patients' OACHs were removed completely, 1 patient's OACH was partly removed, and 3 patients underwent pure decompression of the optic nerve. Fourteen patients gained improved or stable BCVA after surgery. Three patients showed postoperative vision decline, and 1 patient had no light perception after surgery.

**Conclusions:**

Endoscopic surgery is an effective surgical technique for the treatment of benign tumors in the orbital apex. It is necessary to strictly select patients and fully evaluate the benefits and risks of tumor completely or partly removed.

## 1. Introduction

The orbital apex tumor (OAT) is a unique type of orbital tumor due to its tricky location in the orbit that involves the most posterior portion of the orbit and has numerous adjoining critical neurovascular structures [1]. Most OATs are identified in the retrobulbar muscle cone, predominantly in the lateral aspect of the intraconal space. Although they are often benign, even a small tumor in this area can cause significant morbidity, with symptoms including orbital pain, headache, and neuroophthalmologic symptoms such as ptosis, ophthalmoplegia, and loss of vision due to compressive optic and/or cranial neuropathy through compression of nerves and vessels [2, 3]. OATs mainly include cavernous hemangioma, meningioma, schwannoma, neurofibroma, and fibrous tumors [4, 5], which have different characteristics and different imaging findings on CT or MRI. Take cavernous hemangioma as an example; its characteristics are clear: it is the most common orbital lesion in young and middle-aged adults, comprising 7% of primary orbital masses; it usually occurs in patients who are 30–50 years old; it is seen in females 60–70% of the time. They are low-flow vascular malformations that are well circumscribed and have a capsule. They most commonly occur along the lateral aspect of the retrobulbar intraconal space and uncommonly involve the orbital apex [6]. Nevertheless, it should be differential diagnosis includes schwannoma, meningioma, hemangiopericytoma, and lymphoma and sometimes it cannot be distinguished before being surgically taken out for pathology confirming [7]. Serious damage to vision and inability to accurately determine the tumor categorization requires early surgical intervention. And meningiomas are the most common optic nerve sheath tumor in adults and are more commonly seen in women [8, 9]. Progressive painless vision loss and a normal appearing optic disc is the mainly symptoms. Treatment is difficult as surgery is associated with significant visual morbidity. On CT and MRI imaging, there are several common imaging characteristics of optic nerve sheath meningiomas which can help diagnose schwannoma, neurofibroma, and fibrous tumors. The clear diagnosis of schwannoma, neurofibroma, and fibrous tumors also requires postoperative pathological examination.

Traditional orbital apex surgery techniques include extended lateral orbitotomy, medial transconjunctival/transcaruncular orbitotomy, and frontotemporal craniotomy. However, these surgical approaches are difficult and particularly challenging in tumors located inferior to the optic nerve; they can even damage the optic nerve, muscles, and blood vessels in the orbit apex [10]. In recent years, transnasal endoscopic procedure has been described for resection of intraconal orbital tumors. In contrast to the conventional orbitotomy, transnasal endoscopy is convenient to expose the medial intraorbital structures, the orbital apex, and the optic canal, providing a safer, less invasive approach [11].

Reports on the excision of small, benign, medially located lesions have also been published [12–23]. For tumors found within or external to the muscle cone, transnasal endoscopy is usually the favored approach for those located in the nasal side of the optic nerve; otherwise, it could be risky for those located on the temporal side of the optic nerve. However, even excellent transnasal endoscopic surgery technique cannot completely avoid the risks of surgery, especially when the tumor is adjacent to the optic nerve. A global multicenter clinic trial in 2015 revealed that 65.2% patients had visual impairment after endoscopic endonasal resection of an orbital cavernous hemangioma [24].

Given that benign tumors are not fatal, this risk makes it difficult for surgeons to decide when and whether to execute a complete or partial resection of the tumor or to conduct a simple optic nerve decompression. In this article, we take the most common type of OAT, cavernous hemangioma, as an example [25, 26], and we retrospectively analyzed the therapeutic effects of transnasal endoscopic surgery on OATs to further discuss the challenges of transnasal endoscopic surgery in the treatment of OATs and to tentatively discuss possible standards for patient selection, surgical planning, and surgical techniques to further consider surgical choices and intraoperative precautions.

## 2. Methods

We reviewed the medical and surgery records of 18 patients who underwent transnasal endoscopic resection after a diagnosis of uniocular cavernous hemangioma by senior ophthalmologists (Professors Rong Lu and Jianbo Shi) at Zhongshan Ophthalmic Center (ZOC) from March 2016 to May 2020. The clinical data—including presenting symptoms, onset time, radiologic images, surgical approaches, tumor sizes, complications, and outcomes—are summarized in Table 1. Diagnoses of excised neoplastic tissues were confirmed by pathologists. The necessity for surgical treatment was indicated by clinical symptoms attributable to the orbital apex lesions, such as visual decline, diplopia, exophthalmos, ophthalmodynia, or headache. Ethical approval and patient consent were obtained before the surgical procedures were carried out, and the procedures adhered to the tenets of the 1964 Declaration of Helsinki.

### 2.1. Surgical Procedures

**Figure 1:**
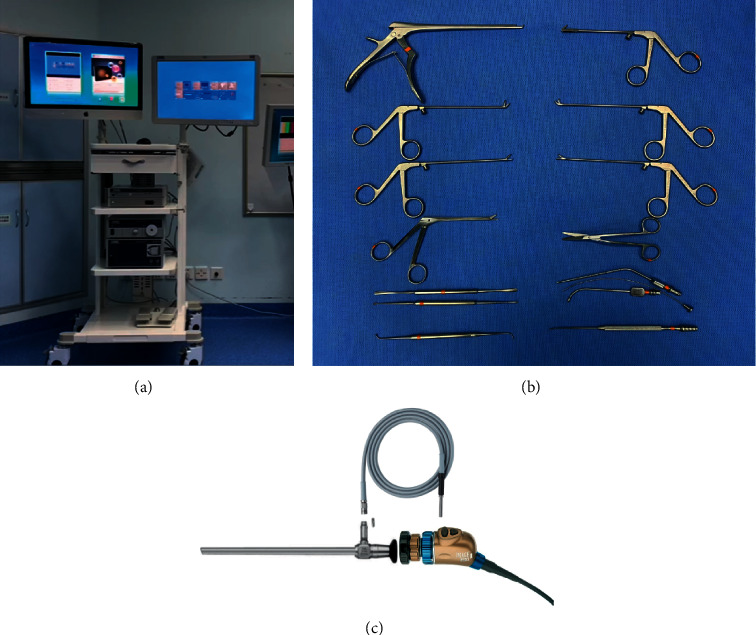
Zero-degree, 4 mm diameter rod-lens rigid telescope system and microsurgical instruments.

All surgeries were conducted under general anesthesia using transnasal endoscopy. The patient was placed in a supine position, with the head tilted to the right and slight chin adduction. Local anesthesia of the nasal mucosa was achieved using a mixture of 1% lidocaine and 0.1% epinephrine. The operation was performed using a zero-degree, 4 mm diameter rod-lens rigid telescope (Karl Storz, Germany) and microsurgical instruments (Figure 1). Following resection of the unciform process, the anterior ethmoidal cells were removed with cutting forceps, and the basal lamella of the middle nasal concha was removed to reveal the ethmoidal sinus. Removal of the middle nasal concha may be necessary in some cases to enhance operative access. Dissection was carried out posteriorly until the sphenoid sinus was reached, and anterior sphenoidectomy was performed to expose the sphenoid sinus roof. The sphenoidal sinus was opened to abundantly expose the medial orbital wall, and the optic protuberance could be identified in the sphenoid roof. In cases in which the OACH is of greater mass, the interior orbital wall may appear to be dark red or exhibit a protuberance due to pressure. The periorbita and orbital fascia became visible as the mucosa was exfoliated, and the lamina papyracea was removed at the orbital apex. An exit cut slightly smaller than the tumor mass was incised on the orbital fascia, according to the dimension of the tumor estimated by MRI.

We encountered several different situations and adapted our approach to each case. In cases of extraconal OACH, they could be immediately visible as soon as the orbital fascia was incised. Some tumors, such as hemangiomas and schwannomas, are usually well encapsulated in a membrane, which facilitates careful dissection from the surrounding adhering connective tissues. Microdissection of the tumor mass is necessary if blunt dissection is insufficient. Orbital fat and medial rectus muscle must usually be excised or forced into the orbital cavity using brain cotton pieces or a brain spatula before the encapsulating membrane is detached from adjacent tissues. On the other hand, where the intraconal OACH is found between the optic nerve and medial rectus muscle, the muscle is lifted upward or pushed downward to expose the tumor.

Detachment should start from proximal to distal of the surgeon. The traction of the detached tumor to the nasal side should be guided by microforceps and further reveal the tumor mass deep in the cavity. Incision with microsurgical scissors is necessary if the tumor adheres tightly to the muscles or other tissues.

In some cases, the tumor mass tightly adhered to the optic nerve, was obstructed by the optic nerve, or even could not be observed. Given the high risk of complications, we chose pure optic nerve decompression instead of tumorectomy.

**Figure 2:**
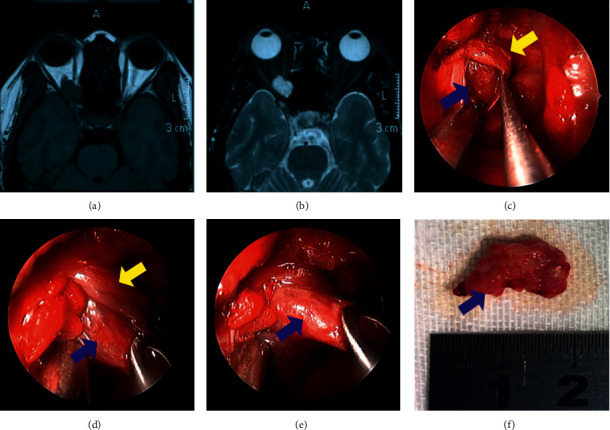
Magnetic resonance imaging of a patient with cavernous hemangioma of the orbital apex and surgical removal process. (a) T1-weighted images showed an isointensity mass in the right orbital apex. (b) Axial T2-weighted images showed a high-intensity mass. (c–f)The surgical removal of the cavernous hemangioma: the yellow arrow indicates the muscle and the blue arrow indicates the tumor.

During the dissection, attentive observation of the pupil of the operated eye was required; a dilated pupil during the procedure may indicate optic nerve injury due to mechanical damage or vascular compromise. In the case of dilated pupils, tractions were released immediately, and precise dissection of the OACH was relocated. In cases of significant adherence to the lateral aspect of the OACH, the surgery was terminated or the OACH was partially resected and sent for histological examination. Hemostasis was carefully performed, and reconstruction of bone defects in the medial orbital wall was not required. The nasal cavity was gently packed only with bioresorbable solution (NasoPore®, Stryker, MI, USA) (Figure 2).

### 2.2. Postoperative Management

Intravenous methylprednisolone (80 mg) and intravenous broad-spectrum antibiotics were given to the patient daily for 3 days after surgery. Nose-blowing and strenuous activity were prohibited within the first month after surgery. Endoscopic examination of the ethmoidal-sphenoidal sinus and a wound surface check were performed for each patient at 2 to 4 weeks after surgery. Postoperative examination was compulsory at week 1, week 2, week 4, month 3, and month 6, and subsequent examinations were carried out upon patient request.

## 3. Results

All the patients' medical information is listed in Table 1. There were 18 patients, 7 males and 11 females, with a mean age of 49.9 ± 12.6 years (range: 26 to 70 years), and the time from the discovery of the OACH to the operation was 16.6 ± 22.8 months (range: 1 to 84 months). All 18 patients had a unilateral tumor. Among the 18 cases, 13 were located in the right orbit and 5 were located in the left orbit. The main clinical symptoms included visual decline (10/18), proptosis (8/18), ophthalmodynia (5/18), diplopia (3/18), headache (3/18), and tinnitus (2/18).

Sixteen patients with OACH underwent purely endoscopic transnasal surgery. In the other 2 patients, the tumor was obscured and tightly adhered to surrounding tissues; therefore, a transcutaneous or transconjunctival surgical approach was adopted to assist the resection. During the surgery, 14 patients' OACHs were removed completely, all of which had membranes and distinct borders, and in 1 patient the OACH was partly removed; it was not well defined and had no membrane. On the other hand, 3 patients underwent pure decompression of the optic nerve.

Unfortunately, 3 patients showed postoperative vision decline, and one patient had no light perception after surgery. We found 1 of the 3 patients had dilated pupils during the surgery; intravenous methylprednisolone (1,000 mg) was given immediately and for 2 days after the surgery, but the BCVA did not improve.

Nasal bleeding was found in 8 patients and spontaneously stopped within 48 hours after surgery. One patient claimed to have postoperative headache, which was resolved within 1 month. All 18 patients' medical data are displayed in Table 1.

## 4. Discussion

As a special subtype of orbital tumors, OACHs are rare but can seriously and adversely affect visual function [15]. Surgical excision is the best strategy to treat the patient and is usually performed as an external orbitotomy with or without osteotomy [10, 27, 28]. However, medial and inferior orbital lesions, especially those at the orbital apex, are extremely difficult to reach via traditional orbitotomy. The challenge is that, during a traditional orbitotomy, many crucial apparatuses must be taken into consideration, including the optic nerve, the extraocular muscles, and the blood vessels in the orbital apex. Moreover, the accessible cavity for operation is very limited.

Kennedy et al. [29] first reported that transnasal endoscopy could be used for optic nerve decompression of thyroid-associated ophthalmopathy, and ever since this report, this approach has been adopted by many practitioners as their preferred treatment strategy. In 1999, the first case of transnasal endoscopy in the removal of an orbital cavernous hemangioma was reported by Herman et al. [30]. In recent decades, transnasal endoscopy has become more popular, yet only limited cases of tumor removal at the orbital apex have been reported.

Unlike other surgical operations, ophthalmological operations require surgeons to work around the lesion within a very small cavity, and the most significant difficulty is insufficient lighting and lack of an accessible field. On the other hand, there are several advantages of endoscopic transnasal orbitotomy. Endoscopy can overcome the problem of visualization deficiency in traditional orbitotomy by providing good visual aids for surgeons, including illumination and magnification to eliminate blind and shading regions, which are vitally important for operations in a limited cavity like the orbital apex.

In particular, intraoperative navigation and computer-assisted surgery (CAS) is widely used in orbit and orbitofacial surgery in recent years. Navigation helps in accurate target localization and establishing tumor margins for resection, CT angiographic guidance can help avoid vascular injuries during tumor excisions and lesion localizer software can be used to separately mark the lesion, optic nerve, and vessels using colour coding [31, 32]. This greatly assists the surgeon and further reduces the risks and difficulties of endoscopic surgery.

In our cases, the telescope used was only 4 mm in diameter, and it could easily access the operation field through the ethmoid sinus and sphenoid sinus, bypassing obstacles such as the eyeball and orbital edge and eventually reaching the tumor for resection. Moreover, operation efficiency with built-in tools was enhanced by endoscopy, and the use of other surgical instruments, such as brain retractors or spatulas, was reduced during traction of loose tumor or tissues off-side to clear the operation field. Endoscopy proved capable of accomplishing the tricky tumor resection task at the orbital apex with minimal disturbance to neighboring structures, such as the optic nerve, the extraocular muscle tissues, and the blood vessels.

Meanwhile, we also observed certain limitations of the endoscopic transnasal approach. Ophthalmic surgeons usually plan operation strategies on the basis of medical imaging; therefore, preoperative imaging to locate and characterize the tumors by CT or MRI is extremely crucial before a treatment decision is made. For tumors found within or external to the muscle cone, transnasal endoscopy is usually the favored approach for those located in the nasal side of the optic nerve. Otherwise, it can be risky for those located in the temporal side of the optic nerve. Endoscopy can be challenging for tumors that require complicated surgical details—such as hemostasis, suturing, or exfoliation for separation—because only limited surgical instruments can enter the orbital cavity via ethmoid sinus. This technique is also favored for simpler tumor resections of cavernous hemangioma, schwannoma, and simple cysts with low hemorrhaging risk. However, surgeons must carefully consider strategies for tumors that are prone to excessive bleeding, such as venous vascular malformations, multiple tumors, and tumors that have massive nourishing blood vessels. It must be noted that whenever an endoscopy is performed on a potentially vascular orbital lesion, especially in the orbital apical mass, a backup external or open plan must be prepared, which should involve an oculoplastic surgeon or ophthalmologist to ensure the complete removal of the lesion with minimal morbidity.

In our experiences in orbitotomy, orbital fat tissues or surrounding muscles could be interruptive during operation. As described in our surgical methodology, the muscles were lifted upward or pressed downward, and the orbital fat was forced aside by spatulas or other tools or sometimes excised for complete exposure of the tumor mass [19]. However, in some cases, the orbital apex tumor was found “hidden” in the surrounding tissues.

Other than less displacement of orbital structures, less postoperative complications was another important way in which transnasal endoscopy is preferred over traditional external operations for the treatment of orbital apex tumor. The complications of the latter include visual deterioration, motility deficits, and ptosis, and it usually takes few months for patients with postoperative motility deficits and ptosis to recover [33]. On the other hand, rates of deteriorated vision, motility deficits, and ptosis were much lower for patients who were treated with endoscopic operations [24, 34]. We found easier operation access and better postoperative outcomes were associated with early diagnosis and prompt treatment (i.e., shorter symptomatic duration).

In some patients in our case series, orbital apex tumor of greater mass was associated with latent diagnosis, and they were often found firmly adherent to apical structures, causing tumor resection to be more challenging, and the postoperative BCVA recovery was often less effective, and vision loss could be associated with one patient who even gets no light perception after surgery. Therefore, the location and characteristics of the tumors were crucial determining factors for postoperative complications and unfavorable prognoses.

Given that, among OATs, benign tumors are the majority, if these patients do not receive surgery, their vision often declines gradually, and whether it is worthwhile to bear the risks of surgery depends on the patient's decision and the doctor-patient communication. This is also determined based on the conditions and specific circumstances of each patient and cannot be generalized. Therefore, when doctors choose which patients can receive endoscopic orbital apex surgery, in addition to fully assessing the tumor's condition, they must also fully consider each patient's situation, especially for patients with normal or remaining visual functions. During the operation, when the tumor is found to be closely attached to the optic nerve, the surgeon should also choose to partially remove the tumor or to just perform optic nerve decompression surgery if appropriate so that the patient can benefit the most from the balance of risks and rewards.

## Figures and Tables

**Table 1:** Clinical characteristics and surgical outcomes of patients with OACH involving orbital apex.

No.	Sex	Age (years)	Main symptoms	Duration of symptom (months)	Tumor location^1^	Tumor size (mm)	BCVA		Tumor removal
							Preop	Postop	
1	F	54	Vision decreased, diplopia	36	OD; inside; inferior medial	21 × 11 × 10	20/20	20/16	Completely
2	M	33	Vision decreased; eyeball prominent	84	OS; inside; inferior lateral	29 × 19 × 21	20/25	20/63	Completely
3	F	44	Vision decreased	12	OD; inside; superior medial	14 × 12 × 15	20/100	20/32	Completely
4	M	53	Tinnitus	0.5	OD; outside; inferior	16 × 25 × 13	20/20	20/16	Completely
5	F	63	Vision decreased, eye pain	3	OD; outside; medial	25 × 13 × 45	20/32	20/20	Completely
6	F	46	Tinnitus	0.6	OD; outside; inferior medial	13 × 10 × 9	20/20	20/16	Completely
7	F	48	Vision decreased, headache	0.5	OD; outside; medial	23 × 13 × 17	20/25	NLP	Completely
8	M	60	Eye pain, headache	2	OS; inside; inferior medial	15 × 10 × 10	20/25	20/20	Completely
9	F	59	Vision decreased, headache, diplopia	60	OD; outside; inferior medial	20 × 16 × 18	20/25	20/20	Completely
10	F	62	Eyeball prominent, swelling and pain	12	OS; inside; inferior medial	25 × 22 × 15	20/25	20/25	Completely
11	M	51	Vision decreased	6	OD; outside; inferior medial	13 × 12 × 12	20/333	20/200	Completely
12	F	31	Eyeball prominent	24	OS; inside; inferior medial	25 × 22 × 16	20/25	20/25	Completely
13	F	63	Eyeball prominent	12	OD; inside; inferior medial	32 × 24 × 21	20/80	20/333	Completely
14	F	55	No obvious symptom	6	OD; inside; inferior medial	17 × 09 × 08	20/80	20/80	Completely
15	M	28	Eyeball prominent	2	OD; inside; superior medial	18 × 117 × 15	20/16	20/32	Partly
16	F	70	Vision decreased, eyeball prominent	1	OD; inside; superior medial	19 × 17 × 13	20/32	20/32	Optic nerve decompression
17	M	53	Eyeball prominent, vision decreased, diplopia	36	OS; inside; inferior medial	24 × 22 × 19	20/50	20/40	Optic nerve decompression
18	M	26	Vision decreased, eyeball prominent	1	OD; inside; superior medial	20 × 18 × 15	FC/20 cm	20/1000	Optic nerve decompression

^1^Tumor location includes information on lateral of the eye with lesion, whether the lesion was inside or outside of the muscle cone, and the relative position to the optic nerve.

## Data Availability

Research data can be found in Zhongshan Ophthalmic Center clinical case database.
